# Undergraduate Nursing Student Perceptions of Clinical Training Approaches: A Quasi-Experimental Study

**DOI:** 10.3390/nursrep15020047

**Published:** 2025-01-31

**Authors:** Kholoud Hardan-Khalil, Ahlam Jadalla, Cathleen M. Deckers, Christine B. Costa

**Affiliations:** School of Nursing, California State University, Long Beach, CA 90840, USA; aj.jadalla@csulb.edu (A.J.); cathleen.deckers@csulb.edu (C.M.D.); christine.costa@csulb.edu (C.B.C.)

**Keywords:** clinical education, nursing, prelicensure, preplanning, reflective care

## Abstract

Background/Objectives: Undergraduate nursing students report encountering significant challenges when they perform preplanning for clinical days. The literature lacks evidence regarding this educational model for clinical training, yet faculty continue to use it despite the lack of evidence that supports it. This study explored undergraduate nursing students’ perceptions of their preclinical training activities. Methods: A quasi-experimental, after-only, nonequivalent control group design was employed at a public nursing school in an urban setting. A total of 110 undergraduate nursing students enrolled in an advanced medical–surgical course. Participants were divided into two groups based on their preparation approach for clinical practice. Data were collected using a paper-and-pencil survey at the end of the course’s clinical rotation. The survey comprised three sections: (1) sociodemographic information, (2) the nursing clinical education tool (NCET) developed for this study, and (3) two open-ended questions focusing on the pros and cons of preplanning and reflective care approaches. The responses were analyzed and compared using a nonparametric two-independent-samples Mann–Whitney U test. Results: The findings indicated that students in eight out of ten nursing clinical education survey categories favored the reflective care approach. No differences were found between groups concerning class grade point average (GPA), the National Council Licensure Examination (NCLEX) passing rate, or standardized tests. Conclusions: The reflective care approach was perceived more favorably than preplanning. Engaging in clinical reasoning strategies requires educators to reconsider how students interact with clinical education. Further research is needed to develop evidence-based methods to enhance the clinical learning experience and promote patient safety.

## 1. Introduction

The complexity of the current healthcare environment presents significant challenges to supervising unlicensed students relative to maintaining patient safety. According to a qualitative study on medication errors made by licensed nurses, staffing, changes in patient condition, and patient turnover contribute to the chaotic nature of clinical nursing practice and, in turn, adverse patient events [1]. Assuring the prevention of unnecessary errors and harm in an ever-changing environment is the key aim of clinical faculty and preceptors.

Nursing relies on clinical training to bridge the gap between theoretical knowledge and professional practice—healthcare in the 21st century demands prepared nurses to deliver safe and high-quality patient care. Traditionally, in the United States, prelicensure nursing students are required to prepare for clinical training by collecting pertinent health data related to patients on the day before the scheduled clinical training. On the other hand, expert voices in nursing education have called for transforming clinical nursing education, such as phasing out from the preclinical preparation approach and adopting reflective care instead [2].

The use of validated educational training models underlies the foundation needed for nurses to think critically and demonstrate competency to meet the needs of a dynamic healthcare system. This presents an ongoing challenge for nurse educators, since the educational model of cognitive apprenticeship training for nurses has remained the same and untested for decades [3,4]. A systematic review concluded that insufficient evidence exists to support the traditional clinical training module [5]. Given the complexities of today’s healthcare environment, there is a need to re-examine how clinical education supports the higher-order thinking and prioritization necessary to maintain patient safety [3,5]. Although preceptor training focusing on teaching/learning strategies, conflict management, facilitation, and assessment/evaluation has been surmised to influence perceived learner outcomes positively [6], formal preceptor training has generally been underutilized as a strategy in undergraduate nursing training programs.

### 1.1. Preplanning Approach

Preplanning, the action of going to the clinical setting prior to the day of practice and gathering information about a selected patient, began as an educational strategy in the early 1930s to increase the deliberate practice of connecting theory to practice using the nursing process while reducing learner anxiety [7,8]. Historically, this practice has been highly revered by faculty as a safeguard for patient safety practices. However, more current research has been conducted on the efficacy of the practice and its impact on patient safety [5].

Prelicensure nursing students in their advanced medical–surgical clinical rotation at a state-run Bachelor of Science in Nursing program bear the heavy load of preclinical preparation in acute care units. However, the changes in clinical facilities triggered by the COVID-19 pandemic have led to changes in students’ clinical education in nursing programs nationwide [9]. Based on most clinical facilities’ requests, nursing schools decreased the number of clinical hours and, in many cases, replaced preclinical preparation with reflective care. Preclinical preparation requires physically being at the facility, long hours of collecting patient information, and then preparing the care plan for the next day. Reflective care allows nursing students to reflect on the clinical experiences they were engaged in during their clinical practice.

A self-report survey study revealed that 55% of students felt that preplanning before going to clinicals contributed positively to their safety as practitioners and knowledge related to patient care [8]. At the same time, most students (71%) reported an increased level of anxiety and a decreased amount of sleep related to the preplanning process, which was perceived to impact their patient safety practices negatively [8]. Students additionally reported that preplanning activities were further complicated by class schedules, transportation considerations, and access to medical records at the clinical sites, which added to the activity’s length [8]. Hospital service partnerships have discouraged preplanning practices because of their impact on the in-patient clinical setting. Many seasoned clinical faculty are reluctant to change the traditional clinical model, fearing that patient care outcomes might be negatively impacted despite the need for more literature to support the existing model [5]. Research has indicated that errors could be higher among prelicensure nursing students because of their novice skills and unfamiliarity with the clinical environment [10]. Medication errors are the most prominent types of errors that occur in prelicensure clinical settings, with half going unreported [11,12]. Medication administration represents only one of the complex skills that student learners must be able to master in the clinical learning setting.

### 1.2. Reflective Care Approach

Reflective care is a new approach in clinical nursing education. Rather than requiring students to conduct preclinical preparation before providing care for patients, nursing students are required to reflect on their clinical experiences after engaging in patient care [13,14]. Reflective care has a different philosophy of preparing competent and safe nurses. Reflective care helps nursing students and other healthcare providers better understand the rationale behind the clinical decisions they make in patient care [15]. Moreover, the reflective care approach helps nursing students experience what registered nurses encounter daily to provide care and fulfill patients’ health needs [16].

Upon receiving the work assignment, nurses collaborate with other healthcare providers, apply the knowledge they learned in nursing programs, and utilize the available system resources to improve patient outcomes. Although the research is limited, a study showed that nursing students perceive pre-simulation activities as beneficial [17]. Nursing students reported experiencing high stress and lack of sleep when they engage in preplanning, which requires preparing patient care plans [8].

There is a considerable knowledge gap in best practices in clinical education approaches that ensure quality learning, promote patient safety, and support student wellness. Clinical activities should aim to find innovative, evidence-based learning approaches that prepare students to model clinical practice while more effectively using time [5,18]. This study explored undergraduate nursing students’ perceptions of their preclinical training activities.

## 2. Materials and Methods

### 2.1. Study Aim and Research Question

This study aimed to explore undergraduate nursing students’ perceptions of their preclinical training activities. The research questions were as follows: (1) Do nursing students perceive differences between preplanned clinical training and reflective care approaches? (2) What are the pros and cons of preplanning? (3) What are the pros and cons of reflective care?

### 2.2. Study Design, Settings, and Participants

This study used a quasi-experimental, after-only, nonequivalent control group design conducted at a public nursing school in an urban setting. Nursing students enrolled in the advanced medical–surgical course during the spring and fall of 2023 were eligible to participate. A total of 110 students participated in the study and completed the survey. The sample was divided into Group I (preplanning) and Group II (reflective care). The effect size was adequate for the effect size measurement in all the nursing clinical education tool (NCET) categories.

### 2.3. Instruments

Due to the lack of validated measures that evaluate nursing students’ perceptions of the effectiveness of preplanning experience, the principal investigators (PIs) developed the study paper-and-pencil survey, which consisted of three sections: (1) Sociodemographic information, including gender, age, and ethnicity. (2) The Nursing Clinical Education Survey (NCES), which includes 38 items that measure participants’ responses on a 5-point Likert scale, where 1 = “strongly disagree” and 5 = “strongly agree”. The NCES items were created for this study based on the relevant literature, the advanced medical–surgical course clinical evaluation tool, and the PI’s experience with the student’s challenges. The NCES items were grouped into ten categories. Table 1 includes the categories and items of the Nursing Clinical Education Survey. The NCES was validated by two content experts who reviewed and approved it after minor modifications were implemented. The third part of the survey included two open-ended questions about the pros and cons of preplanning and reflective care. The survey included ten sociodemographic questions and 28 questions about preclinical training activities.

### 2.4. Data Collection

The data were collected using a paper-and-pencil survey. The study was advertised to prelicensure nursing students in the advanced medical–surgical course through the School of Nursing Learning Management System (LMS). The survey was offered to students on the last day of classes after they completed their clinical rotations. Students were instructed to indicate whether they were carrying out preplanning or reflective care in their clinical training, but no participant identification was requested. All students received a full study description and were allowed to ask questions before they signed the informed consent form.

### 2.5. Data Analysis

IBM’s Statistical Package for the Social Sciences SPSS (version 28) for Mac was used to analyze data. Descriptive statistics were used to describe the sample characteristics. Responses to the nursing clinical education questions from the two groups were analyzed and compared using a nonparametric two-independent-samples Mann–Whitney U test. The Mann–Whitney U test was used to compare differences between this study’s two independent groups, since the dependent variable is measured at the interval level and was found to be not normally distributed. The Mann–Whitney U test is instrumental when the sample size is small, or the data do not meet the assumptions required for parametric tests. Two open-ended questions were included to explore the pros and cons of preplanning and reflective care from the student’s perspective. Thematic analysis was used to organize the narrative data collected in the open-ended questions.

### 2.6. Ethical Considerations

Exempt institutional review board (IRB) status was received from the university where this study was conducted. The completion of the survey was voluntary and anonymous. No student identifiers were collected. Although clinical training is required, the study survey was optional. The completed surveys were kept in a locked cabinet in the primary investigator’s (PI’s) office. The PI entered the data into the SPSS software, and the dataset was password-protected on the PI’s computer device.

## 3. Results

### 3.1. Sample Characteristics

A total of 110 nursing students enrolled in the advanced medical–surgical course completed the survey. Of these, 55 students (50%) were enrolled in the accelerated program (Group I—preplanning) and 55 (50%) in the regular-paced one (Group II—reflective care). The students were predominately female (80%) and from different racial/ethnic groups: White (N = 22, 20%), Asian (N = 41, 37.3%), Black (N = 2, 1.8%), Hispanic (N = 29, 26.3%), and multiracial (N = 16, 14.6%). Approximately 85% of students were under 24 years of age.

### 3.2. Nursing Clinical Education Survey

The average mean number of clinical days completed by students in the reflective care group (Group I) was M = 10.20 (SD = 2.2), and in the preplanning group (Group II) was 8.9 (SD = 1.9). It took Group I students an average of 14 h (SD = 7.2) to complete a standard weekly clinical assignment, while it took Group II an average of 18 h (SD = 3.8) to complete the same assignment. Students in Group I reported sleeping more hours before clinical practice day (M = 5.8, SD = 1.2) than students in Group II (M = 3.6, SD = 1.8). All students reported that it took them a long time to complete the disease pathophysiology section; only 50% reported challenges in developing other sections, such as the actual and potential complications and nursing process.

Students’ responses to the nursing clinical education survey showed mixed results. The Mann–Whitney U test showed that students’ perceptions in the two groups (i.e., preplanning and reflective care) significantly differed in eight out of ten Nursing Clinical Education Survey categories, as shown in Table 2. The two groups did not have different perceptions of critical thinking and knowledge application categories.

### 3.3. Open-Ended Questions

Students responded to two open-ended questions and shared their perceptions about the pros and cons of preplanning. Students described the positive aspects of preplanning the day before clinical as it helped them to (1) understand the patient’s condition, (2) create a better understanding of the patient’s current plan of care, (3) establish appropriate priorities and interventions for care the following day, (4) reduce anxiety for the clinical day by closing the knowledge gap prior to the clinical day, (5) enhance critical thinking, (6) create capacity for the ability to discuss patient care with instructor or preceptor nurse, and (7) have more hands-on time with patient care activities and less time on the computer during the day of clinical.

On the other hand, students mentioned the following negative aspects relevant to preplanning activities: (1) mental and/or physical health compromises such as exhaustion and fatigue, extended sitting time, inadequate eating, and inability to perform self-care activities, (2) sleep deprivation on the night before clinical, (3) commuting without adequate sleep was mentioned as a source of safety concern, (4) stress, (5) a lot of work and not enough time, as students perceived the preplanning activity as an unreasonable amount of work to complete the demands of the preplanning portion of the care plan in a short amount of time, (6) preplanning is too time-consuming, as it requires long hours and up to days to complete all assignments within the care planning activity, and (7) focused on completing the assignment for a grade rather than the learning benefits.

Besides their responses on the clinical education tool, this study’s two groups were compared on three other indicators: class grade point average (GPA), a standardized online exam, which is a program that allows nursing students to prepare for the National Council Licensure Examination (NCLEX), and the National Council Licensure Examination (NCLEX)—a nationwide examination for the licensing of nurses in the United States. There was no difference between the preplanning and reflective care groups regarding their NCLEX passing rate, overall class GPA, and the online standardized test.

## 4. Discussion

While the preplanning approach has long been accepted as the preferred approach to preparing students for clinical learning [7], this study’s results indicate a need to consider implementing changes to the design of nursing practicum courses. Students preferred reflective care over the preplanning approach. Students perceived reflective care as a more effective approach to achieving most of their learning outcomes than traditional preplanning. Participant #1 stated “Having the day before clinical be non-stressful and get enough sleep was extremely beneficial. I felt more organized and calmer going into the clinical day”. Participant #2 stated “No preplanning takes so much stress off our clinical days. We can get more sleep before clinical, which is more realistic for the nursing field”.

Reflective care activities were perceived by students significantly more favorably than preplanning in relation to increased confidence in completing clinical assignments, improved sleep, communication with patients and families, ability to apply pathophysiology concepts and connect the patient’s previous and current health history to anticipate potential complications, and the ability to implement patient-centered interventions that respect the culture-specific values, beliefs, and lifestyles of diverse populations. Reflective care activities also helped students demonstrate proper use of patient care technologies and information systems to support safe care, acknowledge their own limits, seek appropriate resources, and demonstrate aptitude for creative problem solving. However, these benefits were reportedly challenged by health concerns, inadequate sleep, anxiety to get the work done without absorbing the meaning, and the dangers of traveling while tired. Participant # 20 stated “At first, I was worried because I thought I wouldn’t be prepared, but I liked not preplanning. I was able to get enough sleep and did not have to worry about completing the care plan the day before. I was energized and able to get through the clinical day without feeling overwhelmed or that I was behind”. Given the results demonstrated by the group that did not have to preplan, it appears that the perceived benefits of the activity do not significantly outweigh the negative aspects. Participant #3 stated “No preplanning taught me to really focus on patient care and get information from the patient themselves through assessment/talking with them instead of relying on the chart”. Students who were in the group that participated in the post-clinical reflective activity reported that their clinical activities were not negatively affected by not preplanning, except in the area of medication knowledge. Participant # 13 stated “Medications are the biggest concern. Depending on the assigned nurse, I may or may not receive 30 min prior to morning meds to look over patient MAR. When I don’t have time, I can’t research about the medications”.

Nursing educators are charged with graduating safe and competent nurses on time by utilizing innovative approaches in nursing education [19]. Current practices in preparing undergraduate nursing students contradict best practices for clinical education and do not enable students to prepare with patient safety in mind. A study suggested that a shared vision with clinical and community partners to create new models for nursing education is key to adopting contemporary changes in nursing education [20]. Decreasing student anxiety is an additional goal that should be strived towards to maximize safe clinical practice. It is well documented that deliberate practice and mastery learning are needed to improve and retain nursing clinical skills [18], and reflective care facilitates deliberate practice.

Nursing students in this study perceived the load of preclinical preparation in acute care units as disruptive to their sleep. Preclinical preparation requires long hours of collecting patient information and designing the care plan. However, students are often challenged further by having to work around staff time and the workflow of the units where they are assigned. It is common that students show up after having prepared for many hours only to find out that the patients they created care plans for have been moved or discharged. While they may have learned from preplanning, they would likely be frustrated when they cannot apply their developed plans. Stressed, lacking sleep, and physically exhausted, these students are more prone to attrition and burnout, which in turn complicate their learning and progress. Researchers have recommended implementing measures to reduce attrition and burnout among students, such as student-led stress reduction support groups. Moran and colleagues believe that eliminating ineffective clinical training practices would promote resilience in nursing students [21].

This study had a few limitations, including a sample size of only 110 participants and limited representation. The self-reporting survey method relied on the students’ recollections about the process of preplanning and reflective practice, and these data could have been impacted depending on the time of the survey. This study piloted reflective care on students enrolled in one course of clinical training; it would be more influential to include students from all undergraduate levels.

## 5. Conclusions

The lack of empirical evidence related to our educational models for clinical training demands that nursing educators investigate alternative ways of engaging students to ensure that students are prepared for the complexity of current healthcare practice [5]. Activities to prepare undergraduate nursing students for clinical rotations need to be examined relevant to the benefits. The traditional practice of preparing for clinical rotations has been altered by our healthcare partners since the COVID-19 pandemic. It is imperative that academic nurse educators take the time to explore alternative activities to meet the educational needs for the clinical preparation of our unlicensed nursing students.

Engagement in clinical reasoning strategies requires educators to think differently about how students engage with clinical education, as simulation-based education has shown us [22,23]. Moreover, Virtual Reality (VR) and Augmented Reality (AR) simulation training would provide realistic hands-on experiences that mimic real-life scenarios. Prelicensure nursing students can practice their skills in a safe environment and receive immediate feedback. Online modules offer excellent access and flexibility to bridge the gap between theoretical knowledge and practical application. This study’s outcomes highlight numerous deficits related to health and wellness associated with the preplanning approach. These findings reflect similar perceptions from a previously completed study [7].

Future research recommendations include further exploring the impact of preplanning activities and their influence on student clinical performance. Additional research studies should explore the impact of innovative clinical preparation activities, such as standardized simulation activities, mentored and structured clinical preparation with faculty guidance, and reflective debriefing of clinical experiences with faculty, as alternative educational methods for clinical preparation and education. There is a need for research to develop evidence-based ways to ensure patient safety and contribute to an effective learning experience for students.

## Figures and Tables

**Table 1:** Nursing Clinical Education Survey’s categories and items.

Category	Item
Assignment completion	Helps me to complete my clinical assignments effectively
	Turn in assignments on time
Critical thinking	Enhances my critical thinking during the clinical day
	Discuss accurate and logical rationale for interdisciplinary plan of care
	Acknowledge own limits and seek appropriate resources
Competency in communication and education	Perform effective and respectful verbal/nonverbal communication
	Utilize communication that minimizes risk across transitions of care
	Produce clear, accurate, and relevant writing and/or charting.
	Utilize communication with patients and families across the lifespan
Patient-centered care	Utilize teaching strategies based on patient’s health literacy
	Use evidence-based knowledge when providing patient care
	Perform a comprehensive assessment
	Implement appropriate interventions based on patient’s needs
	Implement appropriate interventions that reflect an understanding of pathophysiology, pharmacology, and evidenced-based practice
	Implement nursing interventions that respect culture-specific values, beliefs, and lifestyles of diverse populations
	Evaluate plan of care and modify interventions based on findings
	Perform accurate assessment/intervention/evaluation of patient’s pain
Safe and effective patient care	Makes me a safer practitioner
	Helps me develop and implement my nursing care plan
	Establish priorities of care; organize and complete clinical activities
	Incorporate activities that promote patient safety and quality care
	Perform nursing skills safely and effectively
	Follow agency policies and procedures in providing safe, quality care
	Demonstrate principles of infection control and universal precautions
Leadership	Improves the quality of my participation in post-conference discussions
	Take responsibility for own learning
	Demonstrate growth in leadership characteristics
	Demonstrate aptitude for creative problem solving
Informatics	Provides me time on the computer to enhance my informatics skills
	Improves my ability to collect relevant patient information
	Demonstrate proper use of patient care technologies and information
Nursing skills	Enhances my ability to perform psychomotor skills
	Administer medication safely and accurately
Sleep and stress management	Improves the ability to get a full night’s sleep before clinical day
	Decreases anxiety level during a clinical day
Knowledge application	Apply the pathophysiology concepts
	Accurately interpret the laboratory and diagnostic tests findings
	Develop appropriate nursing diagnoses

**Table 2:** Mann–Whitney U test statistics for the Nursing Clinical Education Survey categories.

Category	Group	N	Mean Rank	Mann–Whitney U Test	Sig. (2-Tailed)
Assignment completion	Group 1	55	67.52	851.5	0.000
	Group 2	55	43.48		
Critical thinking	Group 1	55	59.40	1298.0	0.097
	Group 2	55	51.60		
Communication and education	Group 1	55	60.85	1218.5	0.027
	Group 2	55	50.15		
Patient-centered care	Group 1	55	60.25	1251.0	0.050
	Group 2	55	50.75		
Safe and effective patient care	Group 1	55	62.09	1150.0	0.004
	Group 2	55	48.91		
Leadership	Group 1	55	65.36	970.0	0.000
	Group 2	55	45.64		
Informatics	Group 1	55	64.54	1015.5	0.001
	Group 2	55	46.46		
Nursing skills	Group 1	55	71.66	623.5	0.000
	Group 2	55	39.34		
Sleep and stress management	Group 1	55	80.72	125.5	0.000
	Group 2	55	30.28		
Knowledge application	Group 1	55	55.08	1489.5	0.878
	Group 2	55	55.92		

## Data Availability

The raw data supporting the conclusions of this article will be made available by the authors on request.

## Public Involvement Statement

No public involvement in any aspect of this research.

## Guidelines and Standards Statement

This manuscript was drafted against the SURGE (The SUrvey Reporting GuidelinE) for cross-sectional survey research. A complete list of reporting guidelines can be accessed via the equator network: https://www.equator-network.org/ (accessed on 30 September 2024).
